# High-temperature antiferromagnetism in molecular semiconductor thin films and nanostructures

**DOI:** 10.1038/ncomms4079

**Published:** 2014-01-21

**Authors:** Michele Serri, Wei Wu, Luke R. Fleet, Nicholas M. Harrison, Cyrus F. Hirjibehedin, Christopher W.M. Kay, Andrew J. Fisher, Gabriel Aeppli, Sandrine Heutz

**Affiliations:** 1 Department of Materials, Imperial College London, London SW7 2AZ, UK; 2 London Centre for Nanotechnology, Imperial College London, London SW7 2AZ, UK; 3 Department of Chemistry, Imperial College London, London SW7 2AZ, UK; 4 London Centre for Nanotechnology, University College London, London WC1E 6BT, UK; 5 Department of Chemistry, University College London, London WC1E 6BT, UK; 6 Department of Physics and Astronomy, University College London, London WC1E 6BT, UK; 7 Institute of Structural and Molecular Biology, University College London, London WC1E 6BT, UK

## Abstract

The viability of dilute magnetic semiconductors in applications is linked to the strength of the magnetic couplings, and room temperature operation is still elusive in standard inorganic systems. Molecular semiconductors are emerging as an alternative due to their long spin-relaxation times and ease of processing, but, with the notable exception of vanadium-tetracyanoethylene, magnetic transition temperatures remain well below the boiling point of liquid nitrogen. Here we show that thin films and powders of the molecular semiconductor cobalt phthalocyanine exhibit strong antiferromagnetic coupling, with an exchange energy reaching 100 K. This interaction is up to two orders of magnitude larger than in related phthalocyanines and can be obtained on flexible plastic substrates, under conditions compatible with routine organic electronic device fabrication. Ab initio calculations show that coupling is achieved via superexchange between the singly occupied a_1g_ (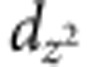) orbitals. By reaching the key milestone of magnetic coupling above 77 K, these results establish quantum spin chains as a potentially useable feature of molecular films.

## Abstract (web summary)

Molecular semiconductors are promising candidates for spintronics applications but they often suffer from low magnetic transition temperatures, usually below the boiling point of liquid nitrogen. Here, the authors observe high-temperature antiferromagnetism in cobalt phthalocyanine films and powders.

## Graphical abstract

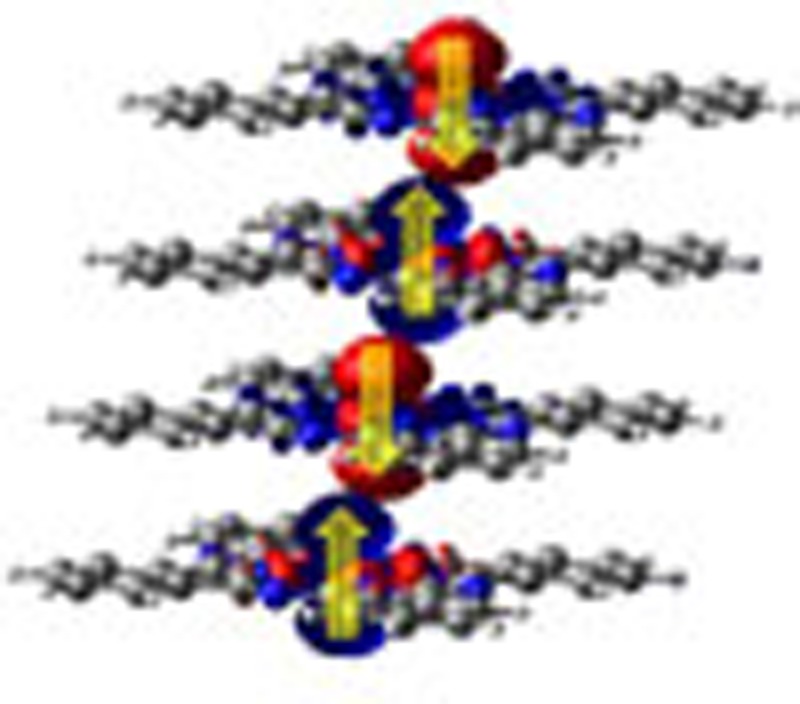

While room temperature magnetism has been observed in specific classes of molecular systems^1^, its combination with semiconducting properties is limited to vanadium-tetracyanoethylene, which has recently been used as a spin injection and detection layer in a spin valve^2^. However, the processing and stability of such materials is delicate^3^,^4^ and a more powerful approach would be to harness the advantages of molecular semiconductors currently used in optoelectronic devices and solar cells^5^. Phthalocyanines (Pcs) are particularly attractive semiconductor candidates, as they are stable small molecules that can host a spin-bearing transition metal ion in a planar conjugated ring; members of this family were among the first molecules to be studied in organic (opto)electronics^6^ and to be recognized as molecular magnets^7^. More recently, there has been growing interest in Pcs in the context of molecular spintronics, and research has concentrated on molecule/electrode interfaces^8^, and the magnetic couplings in thin films, where exchange interactions of up to a few tens of K were observed^9^,^10^,^11^. At the boundary of those fields Chen *et al.* recently investigated magnetic couplings in one to five molecular layers of CoPc on Pb substrates by inelastic electron tunnelling spectroscopy^12^ (IETS). The features of the IETS spectra were assigned to collective spin excitations and a spin flip energy (2*J*) of 18 meV was extracted, corresponding to an antiferromagnetic exchange (*J/k*_B_) of 105 K. These couplings exceed those observed for any undoped Pc film by at least one order of magnitude, but have so far only been detected at the single-molecule level.

Here we show for the first time that CoPc powders and simple thin films grown by organic molecular beam deposition on flexible polymeric substrates, as commonly employed for optoelectronic device fabrication, also display exceptionally strong antiferromagnetic couplings, with *J/*k_B_ ranging between 80 K and 100 K depending on the preparation method. Equally remarkable is that we can essentially switch the couplings off when we slide the molecules away from each other to create another polymorph, available as a powder or an annealed thin film^13^. Our results are rationalized by theoretical calculations showing that the strong spin coupling mechanism is dominated by superexchange between the Co 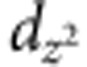 spin orbitals and indicating that the interaction can reach 400 K for molecules stacked co-facially.

## Results

### Structural characterization

**Figure 1:**
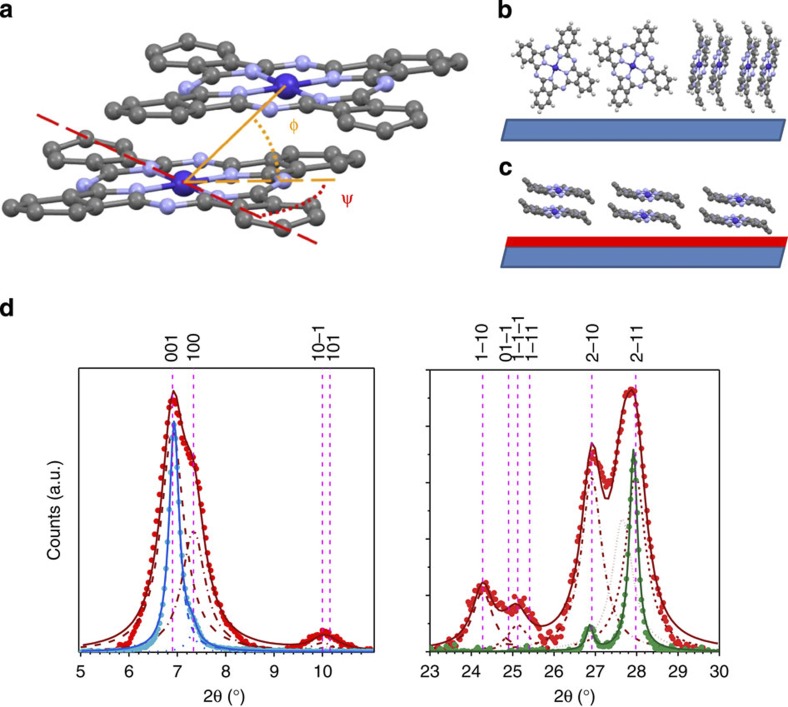
Crystal structure of CoPc powders and films. (**a**) Schematic of the molecular stacking geometry in CoPc crystals. The orange line corresponds to the Co–Co distance and forms an angle *φ* (stacking angle) with the molecular plane. Its projection on the plane (orange dashes) and the Co–N axis (red dashes) define the sliding angle *ψ*. (**b**) Preferential orientation of CoPc molecule in non-templated films, where the substrate (in blue) is aligned with the (001) diffraction plane. (**c**) In the templated case, the CoPc film is grown on PTCDA and the (2–11), depicted, and (2–10) planes are parallel to the substrate. (**d**) Nanocrystalline α-CoPc powder (red dots) with α-CoPc film grown on kapton (blue dots), left panel, and templated α-CoPc film (green dots), right panel. The films on kapton and on PTCDA did not display any signal in the right and left panels, respectively. The main peaks are indexed according to the structure proposed by Ballirano *et al.*^13^ and solid lines correspond to the best fits with Lorentzian peak shapes.

Planar Pcs exist in a range of polymorphs where the molecules stack within columns, and the difference in structure has a strong influence on the sign and magnitude of the magnetic coupling^9^,^11^. The interactions within columns are dominant in determining the magnetic correlations and hence the susceptibility and the magnetic part of the heat capacity; since the Peierls theorem^14^ forbids long-range ordering in one-dimensional systems, the ordering temperatures are, however, determined by weaker inter-column interactions. The orientation of neighbouring molecules within a column can be defined by the stacking (*φ*) and sliding (*ψ*) angles (see Fig. 1a). In the work by Chen *et al.*^12^, the stacking angle (60±3)° is very close to that for α-phase CoPc (65.8°)^13^ and different from that for the thermodynamically stable and more widely studied single-crystal β-phase (42.9°)^13^. Exceptionally strong magnetic couplings might therefore be engineered in a bulk molecular material, since the α-phase can be obtained for small crystallites and, even more usefully for practical applications, in thin films. The X-ray diffraction scans (XRD) of the CoPc thin films and powders are summarized in Fig. 1d and confirm that the materials all adopt the α-phase, as indexed by Ballirano *et al.*^13^ Based on comparison of the peak intensities with the calculated structure factors, it can be deduced that the grains in the powder are randomly oriented. We grew the thin films either directly onto kapton or on a perylene-3,4,9,10-tetracarboxylic dianhydride (PTCDA) first layer, which leads to templating^15^,^16^. As a consequence, the CoPc films are textured preferentially with either their (001) (growth on kapton) or (2–10) and (2–11) (growth on PTCDA) planes parallel to the substrate surface. The molecular orientation relative to those planes is 85.0° for the growth on kapton (Fig. 1b) and 5.7° and 11.6°, respectively, for growth on PTCDA (Fig. 1c).

### Magnetic characterization

**Figure 2:**
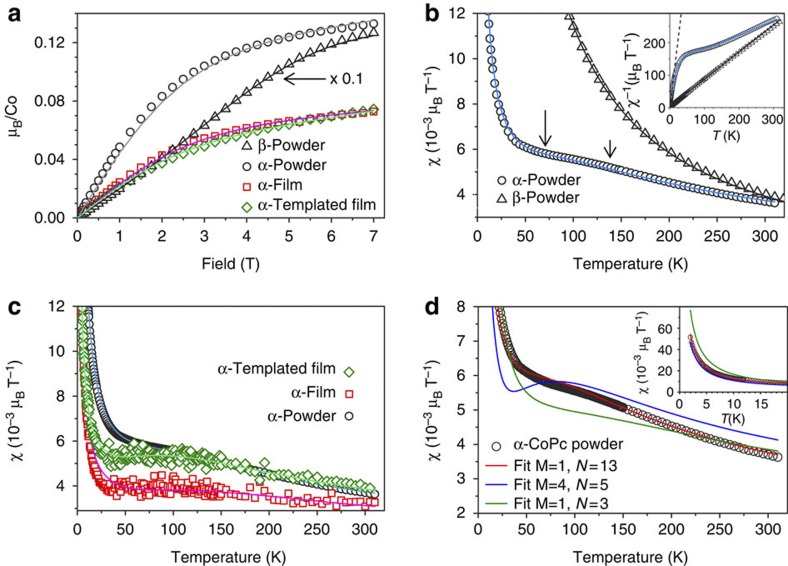
Magnetic characterization of CoPc powders and films. (**a**) Magnetic moment per Co atom as a function of field at 2 K in CoPc samples. The grey, red and green lines represent the fits of the α-CoPc powder, 200 nm film and 200 nm templated film data, respectively, using a Brillouin function scaled by a factor *f* and a *χ*_0_^MH^·*H* correction (see values in Table 1). The black line represents the fit of the β-CoPc data with the Bonner and Fisher model of a Heisenberg spin chain (*S*=1/2, *g*_*z*_=1.9, *g*_*x,*y_=2.9, *J/k*_B_=1.9 K, average of 13 and 14 spin chains). Notice that the β-CoPc has been scaled by 0.1. (**b**) Magnetic susceptibility (*χ*) and inverse magnetic susceptibility (*χ*^*−*1^, inset) of CoPc as a function of temperature. The blue line is a fit of α-CoPc based on equation 1 while the black line is the Curie Weiss fit of β-CoPc powder (8-310 K). The arrows highlight the inflection points. (**c**) Susceptibility of α-CoPc powder and films on kapton. Solid lines are fits using Equation (1). (**d**) Susceptibility of the α-CoPc powder and fits using the finite chain model (Equation (2)) highlighting that the inflection at low *T* (~30 K) and the high temperature susceptibility (see inset) requires contributions from both long and short chains.

Figure 2a depicts the field-dependent magnetization of the CoPc α- and β-phases at 2 K. Although the curve is similar in shape to what would be expected from a paramagnetic spin ½ system, the moment of α-CoPc is substantially suppressed, with values reduced by approximately one order of magnitude. This suggests antiferromagnetic couplings within the majority of the α-phase material, as opposed to the β-phase material, which can be modelled as a weak antiferromagnet with *J/k*_B_=1.9 K (see Methods). The strength of the coupling in the powders is revealed by the temperature-dependent susceptibility, Fig. 2b, where the data for the α-phase do not follow the shape adopted by a paramagnet or a weak antiferromagnet such as β-CoPc. The susceptibility of the α-phase is heavily suppressed, again symptomatic of antiferromagnetism. It displays a broad maximum around *T*_max_=100 K and inflection points at ~50 and 150 K. Bonner and Fisher have shown that this behaviour is characteristic of antiferromagnetic chains and is related to the magnetic exchange interaction through *k*_B_*T*_max_/*J*=1.282 (ref. 17), pointing to an exchange temperature above the boiling point of liquid nitrogen. A transition to a high spin state, that is, to the b_1g_ orbital, would require energies of several eV^18^, and can therefore be excluded. At low temperature, the behaviour of χ^−1^(*T*) is linear, following the behaviour of a paramagnet with a Curie constant corresponding to one-tenth of the Co spins being paramagnetic (see analysis below), consistent with the field-dependent data in Fig. 2a. The change in curvature and transition to a regime dominated by the antiferromagnetism can again be seen between 50 and 150 K.

We now turn our attention to the flexible α-phase thin films. In both types of films, the temperature-dependent magnetization, Fig. 2c, is again characteristic of a strong antiferromagnet with a broad maximum around 100 K. The more pronounced curvature around 50–150 K, especially in the templated case is symptomatic of longer antiferromagnetic chains^17^. Indeed, in an ensemble of finite even and odd spin chains that represents our system, the even chains approach *χ*=0 at low temperatures, highlighting the maximum in susceptibility close to the value of the magnetic exchange, as described above. On the contrary, the odd chains will behave as *S*=1/2 systems, where *χ* diverges as 1/*T* at low temperatures. The *S*=1/2 signal obscures the maximum in *χ* for short chains, but as the chain size increases this contribution diminishes and the inflection points become more clearly defined. The overall shapes of the curves are similar for all samples despite changes in molecular orientation, which is as expected for a spin=½ system, and validates analysis using a Heisenberg model (see Methods).

In order to extract the values of the magnetic coupling, the behaviour of the α-phase has been described as a sum of three terms, namely the susceptibility of an infinite antiferromagnetic chain *χ*_AF_ (ref. 19), a Curie term *C*/*T* and a temperature-independent offset 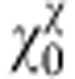:


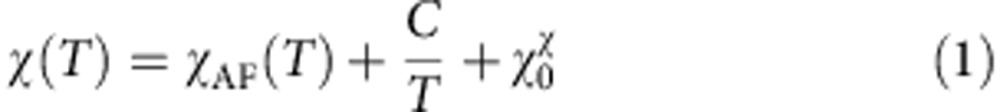


For the powders, the fit yields an antiferromagnetic exchange interaction *J/k*_B_=78 K. The Curie constant *C*=0.0955 μ_B_T^−1^ corresponds to 10.6% of the CoPc molecules behaving paramagnetically with moments *μ*_eff_=2.01 μ_B_. Note that the orbital moment is quenched^18^ and any orbital contributions to the effective moment are entirely accounted for in the *g*-factor value of 2.32 used in the fit (see Methods). The value of 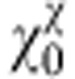 is 1.2 × 10^−3^ μ_B_T^−1^ and the overall positive sign can be rationalized by relatively large positive Van Vleck paramagnetism consistent with low-energy orbital excited states^20^ in addition to the negative diamagnetism of the polyaromatic ring. A similar value for the temperature-independent susceptibility, 
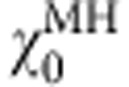
, is also extracted from the field-dependent magnetization, although in that case it is slightly larger^21^. The corresponding paramagnetic fraction, derived from Brillouin fits of field-dependent magnetization after subtraction of 
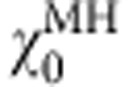
, is 9.6%, again highlighting the consistency of the results. Similar fits were made for the films and are summarized in Table 1. The values of exchange couplings are even higher for the films, with *J/k*_B_=80 and 107 K for the templated and non-templated cases, respectively.

**Figure 3:**
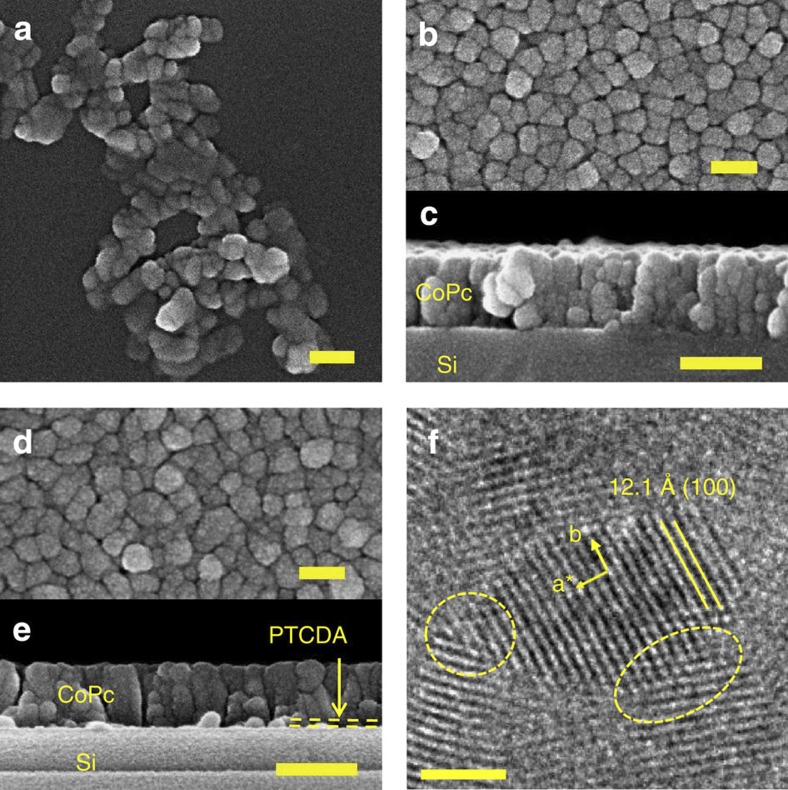
Morphology of α-CoPc films and powders. SEM images of (**a**) nanocrystalline α-CoPc powder, (**b**) top view of a 200 nm α-CoPc film on silicon and (**c**) its cross-section, (**d**) top view of a 200 nm α-CoPc film deposited onto a 20 nm PTCDA layer on silicon with (**e**) its cross-section. (**f**) HRTEM image of a 100-nm-thick CoPc film. The spacing between diffraction fringes is 12.1 Å, compatible with the (100) planes. The ellipsoids highlight disordered regions at grain boundaries. Scale bars are 100 nm in **a**, **b** and **d**, 200 nm in **c** and **e**, and 10 nm in **f**.

**Figure 4:**
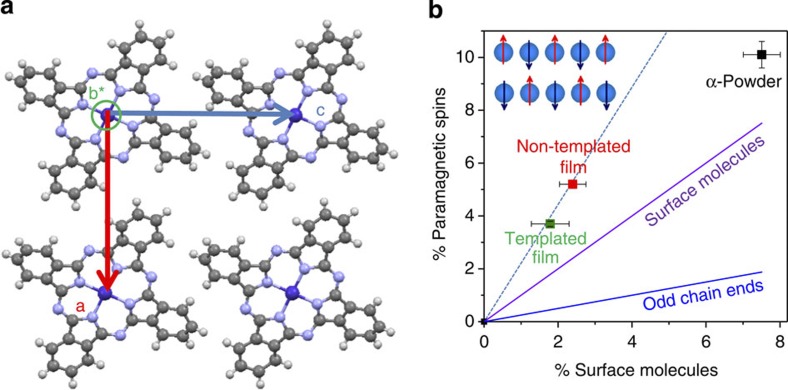
Dependence of magnetism on crystal size. (**a**) Projection of the CoPc unit cell along the b* axis, highlighting that the area of a molecule can be approximated by the product **a·c**. (**b**) Scaling of the fraction of paramagnetic spins in α-CoPc samples with the fraction of surface molecules corresponding to chain ends, and which can be approximated by 3**b**/2*r*, where **b** is the lattice vector (**b**=3.75 Å) and *r* is the particle radius as estimated from the Scherrer equation (Table 1). The fractions of spins and their error bars were determined by averaging the values obtained from the *M*(*H*) and χ(*T*) experiments. The solid lines represent the fraction of paramagnetic spins expected if their origin is either all the surface molecules corresponding to chain ends (slope=1, purple) or only one spin per odd chain (slope=1/4, blue). The dotted line is a fit through the film data and has a slope of 2.2. The cartoon is a schematic representation of the *S*=1/2 ground state for a chain of 5 spins.

The fraction of paramagnetic signal is lower in the films and can be related to the intrinsic structural and morphological properties of the α-phase materials in the three systems studied, rather than to isolated impurities (see Methods). As shown in Fig. 3a–e, α-CoPc forms small spherical particles of typically 23±6 and 55±13 nm diameter in the powder and films, respectively (extracted from top-view images). The cross-section of the films reveals that some particles are somewhat elongated in a direction normal to the substrate, but that their shape is still largely isotropic. To quantify the maximum size of the crystalline domains in the particle, we apply the Scherrer analysis to the XRD scans (Fig. 1d) and the crystal sizes are summarized in Table 1. The resulting values are similar to those found from microscopy, and we can to a first approximation assume that most of the α-phase material is crystalline and forms chains with strong antiferromagnetic couplings. The paramagnetism could arise because of the presence of chain ends at the surface of the grains (see quantification in Methods and Fig. 4a) as suggested by its proportionality to the fraction of surface molecules in the crystallites (Fig. 4b). However, we note that in an ideal system, chains containing even numbers of molecules do not have any resultant spin, and the *S*=1/2 degree of freedom arises solely in odd chains, leading to one paramagnetic spin for every four surface atoms (slope=1/4), considerably fewer than the observed fraction. This discrepancy is most likely due to defects within the chains, in the interiors of the grains, in analogy with what has also been observed for high quality inorganic single crystals^22^, and cannot be accounted for by impurities in the form of Pc molecules containing transition metal atoms other than Co (see Methods).

A closer analysis of the crystallinity of the films by transmission electron microscopy (Fig. 3f) reveals that while the lattice fringes are well-defined throughout the particles, crystallinity is compromised at grain boundaries so that small chains are likely to be present, in addition to long chains in the crystalline core. To conform with this structural configuration, the susceptibility is fitted using a finite chain model (Equation (2)), where the magnetic properties of the chains are calculated using the Bonner and Fisher method^17^ and averaged according to a model chain length distribution P(L) (see Methods). In contrast to the previous analysis (Equation (1)), we have not included a Curie term, as the low-temperature increase of *χ* is intrinsic in finite chains. We adopt the simplest distribution that allows the existence of four characteristic chain lengths (*N*, *N+1*, *M* and *M+1* in Equation (2)), which reflects the expected equal number of odd and even chains in the sample; this is more appropriate than a single chain length model for describing a material that is likely inhomogeneous:


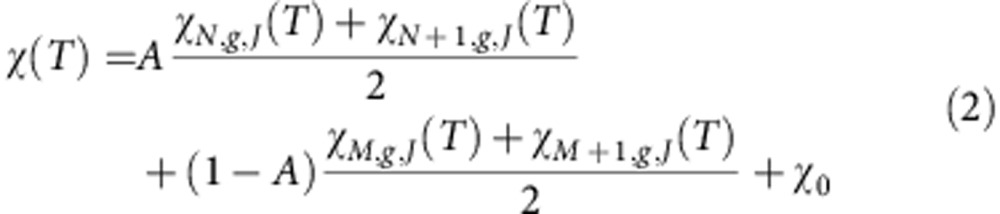


The fraction of paramagnetic spins (*f*) represents the ratio of magnetic moment observed at low temperature and low field to the total expected magnetic moment of uncoupled CoPc molecules. In the absence of defects, this magnetic moment is caused by antiferromagnetic chains with an odd number of spins and an *S*=1/2 ground state. The value of *f* for each fit in the frame of the finite chain model can therefore be calculated using Equation (3).


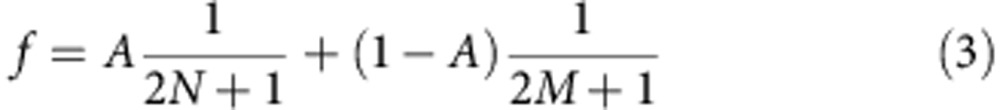


**Figure 5:**
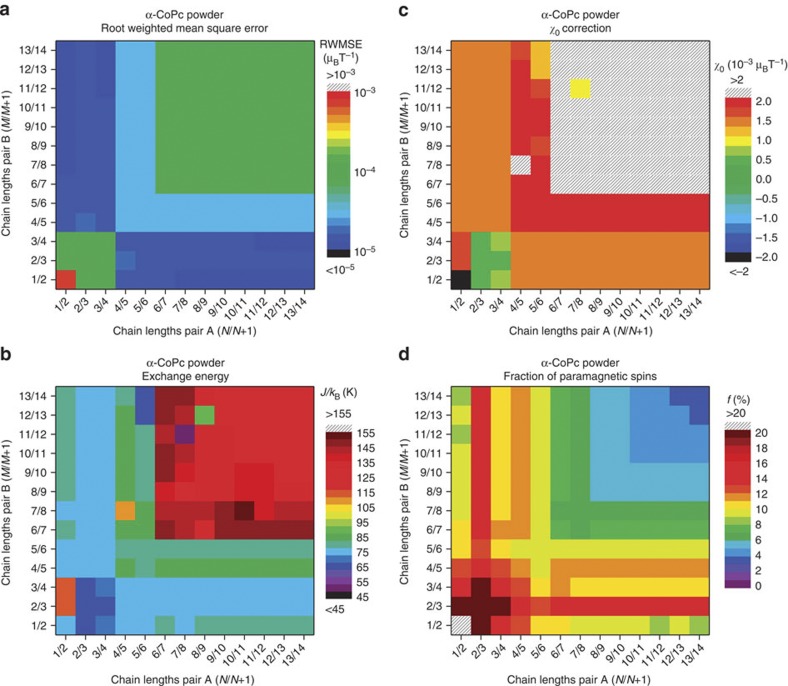
Results of the fit of the α-CoPc powder susceptibility using the bimodal finite chain model. Residuals and values of the fitted parameters are shown in the colour map for all combinations of chain lengths. (**a**) Weighted root mean square error of the fit; (**b**) values of the exchange energy *J/k*_B_; (**c**) values of the temperature-independent correction *χ*_*0*_; (**d**) fraction of paramagnetic spins.

The success of the bimodal chain length approach can be seen in Fig. 2d; fitting of the inflection at low *T* (~30 K) and the high-temperature tail of the susceptibility (see inset) requires contributions from both long and short chains. The result of the analysis for α-CoPc powders for the whole range of chain lengths varying between 1 and 14 molecules is presented in Fig. 5. By identifying regions where the residuals are smallest, we can again deduce that the optimal chain distribution must include both short chains (1–4 molecules) and longer chains (4–14 molecules) in the sample. Within this region of optimal chain distribution, the fit parameters are relatively constant, with an average value of *J/k*_B_, *χ*_0_ and fraction of paramagnetic spins of 73 K, 1.33 × 10^−3^ μ_B_T^−1^ and 11.4%, respectively, as summarized in Table 2. A similar analysis for the films yields exchange interactions of 103 and 79 K for the non-templated and templated films, respectively, and all values are in line with the treatment based on the infinite chain (Equation (1), and Table 1). The presence of the shell of short chains rationalizes why the fraction of paramagnetic spins deviates from the model system described in Fig. 4b. The maximum number of 14 spins corresponds to a chain length of approximately 5 nm. This is below the value for the crystal size derived by the Scherrer analysis of the XRD patterns (Table 1), or the regions displaying lattice fringes in the TEM (Fig. 3f). This discrepancy can be rationalized in two different ways. First, the crystallites may contain defects that are invisible to diffraction techniques (disordered systems do display sharp Bragg peaks as long as the effects of defects on the underlying lattice are short-ranged) even while they limit the spin chains. For example, the variations of contrast along the (100) lattice plane in Fig. 3f could be due to beam damage, but might also indicate either missing molecules or intrinsic displacements of the molecules, both of which could modulate the exchange interactions so as to produce cut-chain magnetic data. Such behaviour has been seen before, even in more ideal inorganic systems^22^. Second, our simulations were limited to a maximum of 14 molecules due to computational constraints. Longer chains could also be present, as above ~10 molecules the shapes of the susceptibility curves converge above ~50 K. In this case, a higher fraction of isolated spins or short chains would be required to compensate for the decrease in the relative number of uncoupled surface spins. In all cases we can however ascertain that the value of 14 molecules derived from the Bonner–Fisher analysis provides a lower bound on the maximum spin chain length in α-CoPc.

### Theoretical analysis

**Figure 6:**
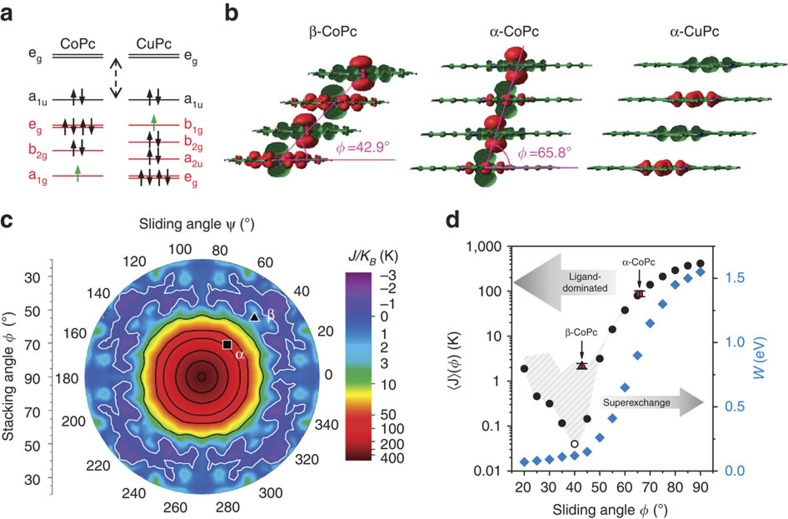
Theoretical calculations rationalizing the dependence of magnetic coupling on structure. (**a**) Energy levels for CoPc and CuPc; the levels with mainly d-orbital contribution are in red, the single spin is symbolized in green, and the dotted arrow represents the HOMO-LUMO gap. (**b**) Spin densities of CoPc in the β- and α-configurations, compared to those for α-CuPc. The iso-surface value for the contour is set to 0.001*e *Å^−3^. (**c**) The contour plot shows the calculated exchange energy as a function of the stacking and sliding angles for CoPc. Note that the height scale is linear from −3 to 3 K, and logarithmic thereafter. Contour lines are drawn at 0, 10, 50 K, and then for every 100 K increment from 100 to 400 K. The points corresponding to the α-CoPc (□) and β-CoPc (Δ) parameters have been highlighted. (**d**) Stacking angle dependence of the theoretical bandwidth (*W*) of the a_1g_ states (azure diamonds) and the azimuthal average of the exchange energy 
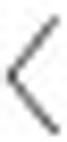
*J*
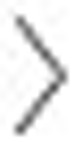
(*φ*) (black circles); for *φ*=40°, 
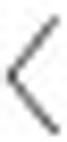
*J*
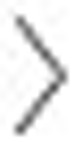
(*φ*) is negative and its absolute value is shown instead (empty circle). The experimental values of *J/k*_B_ in α- and β-CoPc are highlighted with red symbols, with the error bar derived from the standard deviation for values obtained in all films and powders for the α-polymorph, or from the difference between *J*/*k*_B_ extracted from *M*(*H*) or *χ*(*T*) measurements for the β-polymorph. The top of the shaded region represents the maxima of *J*(*φ*) as a function of *φ*. At stacking angles above 50° the height of this region becomes negligible, showing that the spin coupling is nearly independent of the sliding direction. This is diagnostic of the dominant role of the superexchange mechanism at high stacking angles, also shown by the simultaneous increase of the bandwidth *W*. For stacking angles below 50° other exchange mechanisms mediated by the organic ligand become dominant, introducing a marked dependence of *J* upon the sliding direction.

Density functional theory elucidates the mechanism for the exceptionally high magnetic couplings in our CoPc nanocrystals. First, single-molecule calculations yield a spin-½ ground state of ^2^A_1g_ symmetry, in which an a_1g_ orbital derived from the out-of-plane Co 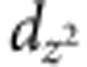 atomic orbital is singly occupied, in contrast to the mainly 
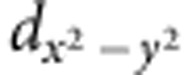
 character of the CuPc singly occupied orbital, see Fig. 6a. The computed spin densities for both CoPc polymorphs and CuPc in Fig. 6b provide intuition as to how the unpaired spins in the *a*_1*g*_ orbitals and the proximity of the metal atoms are key to strong magnetic coupling. The first orbital excited state in CoPc, where one of a degenerate pair of *e*_*g*_ orbitals is singly occupied, is only ≈20 meV above the ground state^18^, thus rationalizing the large Van Vleck paramagnetism. We could not resolve experimentally any contribution from the excited orbital state up to 300 K in the case of the uncoupled spins, as the slope of the susceptibility in β-CoPc was invariant in this temperature range (see inset of Fig. 2b). Even if higher orbital states were populated at 300 K in the α-phase, as has been for example suggested by polarized X-ray absorption spectroscopy^20^, the conclusions of strong antiferromagnetic coupling in α-CoPc would not be affected, as such couplings represent by far the simplest explanation for the difference between the magnetic susceptibilities measured for α-CoPc and β-CoPc. Fixing the inter-planar spacing to that of the α-phase, the electronic structure and the exchange interactions of a one-dimensional chain have been calculated (see Methods) for a wide range of stacking and sliding angles in Fig. 6c and d. The computed exchange interaction of α-phase (β-phase) is *J/k*_B_=85 K (*J/k*_B_=2 K), which is in excellent agreement with the experimental values. The exchange interaction is strongly dependent on the Co–Co distance, which is determined by the stacking angle but only weakly dependent on the sliding angle. This is consistent with the fact that our couplings for the α-phase are similar to those of Chen *et al.,* even though the sliding angles differ by ~52°.

In the co-facial stacking geometry (stacking angle 90°), the computed exchange interaction reaches its maximum at *J/k*_B_ ≈400 K. The variations with structure and composition can be understood in terms of a simple model. The superexchange contribution to the exchange interaction can be estimated as 
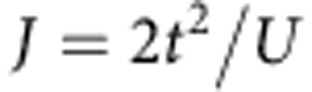
 where the intermolecular hopping integral 
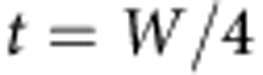
; the bandwidth (*W*) and on-site Coulomb repulsion (*U*) can be derived from the computed band structure. As long as *J* scales as the square of *W*, the mechanism can be described as superexchange, dominated by the d-orbital contributions, and *J* will depend exponentially on the metal–metal distance with weak dependence on sliding angle, as is observed for stacking angles greater than ~45°. The more complex behaviour at smaller stacking angles is due to competition with alternative mechanisms such as indirect exchange^18^, which are sensitive to molecular orbital overlap and therefore show a greater variation of *J* with sliding angle. The change in normalized standard deviation as a function of stacking and the falloff of the bandwidth both qualitatively distinguish between different exchange mechanisms, as shown in Fig. 5d.

## Discussion

We have measured the magnetic properties of α-CoPc in three distinct configurations, namely powder and thin films with two different textures. In addition, we modelled the field- and temperature-dependent magnetization using two approaches, incorporating either infinite chains with paramagnetic contributions or a binomial distribution of chain lengths. The latter reflects the observation by TEM that the α-CoPc grains consist of a shell of disordered molecules around a highly crystalline core. Both methods yield similar exchange interactions, with *J/k*_B_ ranging between 73 and 106 K. While the fraction of paramagnetic contributions is roughly in inverse proportion to the particle size, the exchange interaction remains relatively constant for all α-CoPc samples, highlighting that the strong antiferromagnetism is due to the stacking geometry of the molecules within the crystalline core. This is corroborated by our theoretical analysis and the similarity of our results with those by Chen *et al.*^12^ on CoPc assemblies with almost identical stacking angles to the α-phase.

The observation of magnetism at temperatures above the boiling point of nitrogen, coupled to appreciable hole mobilities in CoPc films^23^ and long spin decoherence times for CuPc^24^, establishes molecular materials as quantum spin chains^25^,^26^ and viable alternatives to inorganics^27^ in the field of semiconductor spintronics. The magnetic centre is embedded in an organic framework, preventing any phase segregation of magnetic dopants, and offering significant advantages for fabrication compared to inorganics; further spin dilution can be easily achieved by coevaporation^28^. The rationalization of the unusually large magnetic coupling in terms of a simple model derived from *ab initio* calculations has established the important role of the out-of-plane 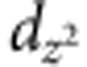 orbital, which is unique to CoPc within the transition metal Pc series, but can be used as a guide to design other macrocyclic complexes with strong interactions. If materials can be created with reduced Co–Co distances, room temperature operation may become a reality.

## Methods

### Sample growth

CoPc (Sigma-Aldrich) was purified once by temperature gradient sublimation to yield the β polymorph. We followed the acid pasting method^29^ to obtain α-CoPc polycrystalline powder. The methods do not efficiently separate Pcs with similar mass. The compositions of the purified powders were therefore verified by inductively coupled plasma mass spectrometry: the level of metal impurities (Cu, Mn, Fe) was below the detection limit (that is, 0.2% on mass), while the error on the absolute mass of Co in the powder (that is, ~1%) provides an upper bound on the fraction of H_2_Pc that may be present. CoPc thin films were prepared using a SPECTROS organic molecular beam deposition system by Kurt J Lesker at a growth rate of 1 Å s^−1^ on flexible Kapton substrates (25 μm thick, Katco Ltd), silicon (100) wafers covered with their native oxide and glass, all held at room temperature, and are 200 nm thick unless otherwise stated. This leads to a preferential orientation of the crystallites with their (001) planes parallel to the substrate, corresponding to molecular planes nearly perpendicular to the substrate. Other CoPc thin films were deposited on a previously grown 20-nm-thick PTCDA templating layer, to orient the molecular plane of CoPc almost parallel to the substrate^15^.

### XRD and quantification of chain ends

The diffraction scans were recorded with a Panalytical X-pert Powder diffractometer, with Bragg-Brentano geometry operated in the *θ*–2*θ* mode and Cu K_α_ radiation (40 kV, 40 mA, nickel filter).

The surface molecules in Fig. 4 are equivalent to the number of chain ends. As the CoPc molecules stack along the lattice vector **b**, the cross-sectional area of a chain can be approximated as the scalar product of the lattice vectors **a** and **c,** and Fig. 4 shows the projection of the unit cell perpendicular to the b* axis. The number of chains in a particle with radius *r* is π*r*^2^/**ac**, while the total number of molecules is 4π*r*^3^/3**abc**. Therefore the fraction of molecules corresponding to chain ends is 3**b**/2*r*.

### Magnetometry

For magnetometry, we used a Quantum Design MPMS-7 SQUID. The CoPc films were measured following previous methods^11^. Powders were loaded in small gelatine capsules. The field-dependent magnetization was measured at 2, 4, 6, 8, 10 and 15 K. The differential magnetic susceptibility of samples was calculated as 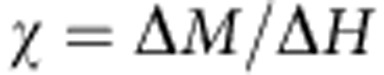, using magnetization measurements at 500 and 1,000 Oe for the powder samples, and 3,000 and 5,000 Oe (still within the linear region of *M*(*H*)) when measuring the films, due to the smaller magnetic moment of the sample. The curves were fitted with the Bonner–Fisher model^17^ using the linear chain Heisenberg Hamiltonian 
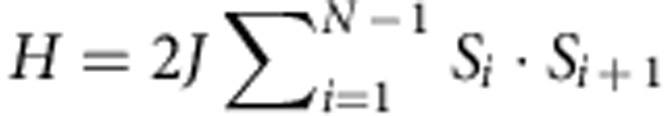
, unless mentioned otherwise. The average value of *g* used is 2.32, extracted from electron spin resonance experiments, and in agreement with the literature value of 2.29 (ref. 30).

### Electron microscopy

The morphology of the samples on silicon was investigated with a LEO 1525 Gemini FEGSEM (5 kV), on samples coated with a thin (10–15 nm) chromium layer. High-resolution images of 100 nm CoPc films deposited on Cu grids were obtained with a JEOL 2010 TEM (200 kV).

### Model distribution of chain lengths in CoPc samples

The model of the chain length distribution in CoPc samples used for finite chain fits of the magnetic susceptibility reflects the core-shell structure of the grains observed by TEM and can be expressed as a bimodal distribution of chain lengths. The probability, *P(L)*, of spin chain length *L* is therefore:


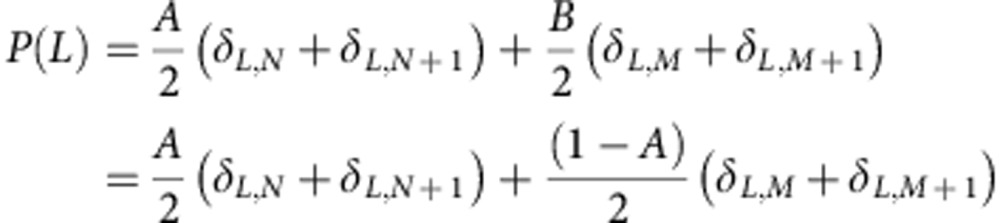


where *δ*_i,k_ is the Kronecker delta.

Since the microscopy does not provide enough unambiguous information to constrain a general *P(L)*, we adopt the simplest model where each region is represented by a pair of adjacent chain length values (*N* and *N+1*, representing the group A of chains, or *M* and *M+1,* representing the group B of chains) occurring with the same probability, in order to reflect the expected equal probability of realizing chains with an odd or even number of spins; the averaging between odd and even chains is necessary due to their different behaviour at low temperature.

For the fitting of the susceptibility data of each α-CoPc sample using Equation (2), the maximum chain length that we could simulate on a desktop computer was 14 spins long. The quality of the fit was evaluated using the root weighted mean of squared errors, where the squared errors are weighted by the density of data points at each temperature. The *J/k*_B_ and *χ*_0_ values were constrained during the fit between 0 and 300 K and −3 × 10^−3^ and 3 × 10^−3^ μ_B_T^−1^, respectively.

### Hybrid exchange density functional theory calculations

Preliminary calculations for the electronic structure of a single CoPc molecule have been carried out using hybrid exchange density functional theory in the Gaussian 09 code^31^. A 6-31G basis set was chosen for all the elements in single molecules and chains. To determine the intermolecular interactions, the electronic structures of one-dimensional periodic chains with two neighbouring molecules per unit cell have been calculated using periodic hybrid exchange density functional theory as implemented in the CRYSTAL 09 code^32^. The Monkhorst–Pack sampling^33^ of reciprocal space was carried out choosing a grid of shrinking factor equal to eight. The truncation of the Coulomb and exchange series in direct space was controlled by setting the Gaussian overlap tolerance criteria to 10^−6^, 10^−6^, 10^−6^, 10^−6^ and 10^−12^ (ref. 32). The self-consistent field procedure is converged to a tolerance of 10^−6^ a.u. per unit cell. To accelerate the convergence of the self-consistent field process, all calculations were performed by adopting a linear mixing of Fock matrices by 30%.

Electronic exchange and correlation were described using the B3LYP hybrid functional^34^ for both the single molecules and molecular chains. The advantages of B3LYP include a partial elimination of the self-interaction error and a balancing of the tendencies to delocalize and localize wave-functions by mixing Fock exchange with that from a generalized gradient approximation. B3LYP as implemented in CRYSTAL has previously been shown to provide an accurate description of the electronic structure and magnetic properties for both inorganic and organic compounds^35^,^36^. The broken-symmetry method^37^ was used to localize anti-aligned spins on each molecule in order to describe the antiferromagnetic state. This approach allows us to calculate the intra-chain exchange interaction as 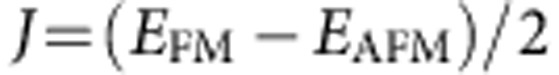 where *E*_FM_ and *E*_AFM_ are the energies of the DFT ferromagnetic state and of the broken symmetry antiferromagnetic Kohn–Sham solution, respectively. In the calculations reported here the structure was not relaxed: the inter-plane distance was fixed and the structure of the isolated molecule was used. For any relaxation to give reliable results a functional would be needed that (unlike B3LYP) accounts correctly for van der Waals interactions. We have shown elsewhere^18^ that such relaxation changes inter-plane distances by at most 8%, in line with the ~5% changes between α- and β-polymorphs determined experimentally^13^, with a corresponding increase in the exchange of about 8% for the α-CoPc phase.

## Author contributions

C.F.H., A.J.F., G.A. and S.H. conceived the study. M.S. grew and characterized the samples, and did the data analysis, which was discussed with all authors. TEM measurements were performed by L.R.F. W.W. performed calculations with supervision from N.M.H. and A.J.F. The manuscript was written by M.S. and S.H., with contributions from all authors.

## Additional information

**How to cite this article:** Serri, M. *et al.* High temperature antiferromagnetism in molecular semiconductor thin films and nanostructures. *Nat. Commun.* 5:3079 doi: 10.1038/ncomms4079 (2014).

## Figures and Tables

**Table 1:** Magnetic data extracted using the infinite chain model.

	***J/k***_**B**_ **(K)**	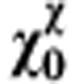 **(μ**_**B**_**T**^**−1**^**)**	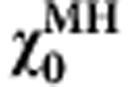 **(μ**_**B**_**T**^**−1**^**)**	***f***^**χ**^	***f***^**MH**^	**Size (nm)**
α-CoPc powder	78	1.2 × 10^**−**3^	3.8 × 10^**−**3^	0.106	0.096	15±1
200 nm α-CoPc film	107	1.1 × 10^**−**3^	2.3 × 10^**−**3^	0.052	0.052	47±7
200 nm α-CoPc/20 nm PTCDA	80	1.2 × 10^**−**3^	4.1 × 10^**−**3^	0.038	0.036	63±18

The exchange energy *J/k*_B_, the temperature-independent correction 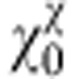 and the fraction of paramagnetic spins *f*^χ^ were obtained by fitting with Equation (1) (Johnston formula). Parameters with the superscript MH refer to the fits on the magnetic moment curves. The grain size was extracted from the XRD peak width.

**Table 2:** Magnetic data extracted using the finite chain fits of the **α**-CoPc susceptibility.

**Chain length range**	**RWMSE (10**^**−5**^ **μ**_**B**_**T**^**−1**^**) average**	**RMSE (10**^**−4**^ **μ_B_T^−1^) average**	***J/k***_**B**_ **(K) average**	***χ***_**0**_ **(10**^**−3**^ **μ_B_T^−1^) average**	***f*** **(%) average**
**Group A**	**min**	**min**	**min**	**min**	**min**
**Group B**	**max**	**max**	**max**	**max**	**max**
*α-CoPc powder*					
	2.10	6.13	73	1.33	11.4
1–4	1.23	4.31	72	1.27	8.9
4–14	2.87	7.53	77	1.37	14.0
					
*200 nm CoPc film*					
	1.64	3.11	103	1.19	6.2
1–8	1.55	2.80	100	1.13	5.2
8–14	1.74	3.36	106	1.22	7.0
					
*200 nm CoPc/20 nm PTCDA*					
	2.83	8.04	79	1.29	5.0
1–8	2.42	6.43	77	1.19	3.7
8–14	3.90	12.2	82	1.54	5.9

The fits were performed using Equation (2). The chain length range defines the chain distributions P(L), defined in the Methods, that result in the best fits (that is, RWMSE <4 × 10^**−**3^  μ_B_T^−1^); RWMSE is the root weighted mean of squared errors, where the squared errors are weighted by the density of data points at each temperature; RMSE is the root mean of squared errors. *J/k*_B_ is the exchange energy, *χ*_*0*_ is the temperature-independent correction, *f* is the fraction of paramagnetic spins predicted at low temperature, calculated with Equation (3).
